# Prevalence of *Blastocystis* sp. and other gastrointestinal pathogens among diarrheic COVID-19 patients in Italy

**DOI:** 10.1016/j.nmni.2024.101228

**Published:** 2024-02-15

**Authors:** Marianna Marangi, Sonia Boughattas, Felice Valzano, Gianfranco La Bella, Rosella De Nittis, Maurizio Margaglione, Fabio Arena

**Affiliations:** a Department of Clinical and Experimental Medicine, University of Foggia, Viale Luigi Pinto, 71122, Foggia, Italy; b Biomedical Research Center, Qatar University, Doha, Qatar; c Istituto Zooprofilattico Sperimentale della Puglia e della Basilicata, Via Manfredonia 20, 71121, Foggia, Italy; d Microbiology and Virology Unit, Ospedali Riuniti, Viale Luigi Pinto, 71122, Foggia, Italy

**Keywords:** COVID-19, Gastrointestinal pathogens, Diarrhoea, Hospitalized patients, Italy

## Abstract

**Background:**

Gastrointestinal pathogens (GPs) contribute significantly to the burden of illness worldwide with diarrhoea being the most common among gastrointestinal symptoms (GSs). In the COVID-19 disease, diarrhoea, could be one of the initial presenting symptoms. However, no data on the potential correlation between diarrhoea-causing pathogens and SARS-CoV-2 infection are available. Therefore, we carried out a 2-years retrospective study aimed to evaluate the prevalence of “classic” GPs among SARS-CoV-2 infected and non-infected patients with diarrhoea in Italy.

**Methods:**

Results of SARS-CoV-2 research from nasopharyngeal and detection of GPs from stool swab samples by Allplex™ SARS-CoV-2 and GI Virus, Bacteria and Parasite Assay were analysed for all patients with diarrhoea referring to Policlinico Ospedaliero Universitario, Foggia, (Italy) from February 2022 to October 2023.

**Results:**

Out of the 833 involved patients, 81 (3.9%) were COVID-19 positive, while 752 (90.3%) were COVID-19 negative. Among COVID-19-positive patients, 37% (n = 30/81) were found positive for one or more GPs with a higher prevalence of protozoan parasites (18.5%) (*Blastocystis* ST1-ST4 subtypes, *Dientamoeba fragilis* genotype I), followed by bacteria (7.4%) (*Campylobacter* sp., *Salmonella* sp.). Viral pathogens were more frequent among COVID-19 negative patients (Adenovirus, Norovirus). Among GPs, *Blastocystis* ST3 subtype was the most prevalent registered in the 16% of patients (p = 0.0001).

**Conclusions:**

Based on obtained results, a likely interaction between the classic GPs and SARS-CoV-2 infection can be speculated, driven by protozoan parasites. Moreover, these results also provide baseline data to understand more deeply *Blastocystis* sp. role in this scenario of dysbiosis, particularly in those cases of SARS-CoV-2 co-infection.

## 1 Introduction

Gastrointestinal infections (GIs) contribute significantly to the burden of infectious diseases illness worldwide [1,2]. Centre for Disease Control and Prevention (CDC) estimates that each year about one billion people worldwide, especially children and elderly, are infected with at least one of the most prevalent species of intestinal pathogens, including virus, bacteria and protozoan parasites, with diarrhoea being the most frequent among gastrointestinal symptomology [2]. While GIs are common in both high-income and medium/low-income countries, they are associated with different risk factors depending on the background. Indeed, within medium/low-income countries, illness is often linked to the lack of clean water and sanitation-related factors, whereas within high-income countries GIs are more often associated with foodborne transmission, seasonal prevalence as well as traveling [1].

The 2019 coronavirus disease (COVID-19), caused by the severe acute respiratory syndrome coronavirus 2 (SARS-CoV-2), has become a global pandemic with cases spreading rapidly throughout the world [3]. While fever and cough were the most frequently symptoms associated with COVID-19, the gastrointestinal manifestations have been reported for 32–61% of individuals with SARS-CoV-2 infection [4]. In particular, diarrhoea has been reported from 4 to 34% of individuals in cohorts from China [5], from 12 to 34% in cohorts from USA [5] and approximately from 10 to 20% within hospitalized patients from Europe [6]. A likely mechanism underlying the occurrence of gastrointestinal symptoms in COVID-19-positive patients is the presence of Angiotensin Converting Enzyme 2 (ACE2) receptors on gut epithelial cells, a well-described target used by SARS-CoV-2 for host cells binding and entry [7,8]. Moreover, the diarrhoea and malabsorption caused by SARS-CoV-2 infection may be due to the dysregulation of intestinal ion transporters [9], leading to inflammation and gastrointestinal symptoms [10]. The entry of inflammatory cells, including neutrophils and lymphocytes, into the intestinal mucosa alters the gut microbiota composition [11]. Thus, this imbalance gut microbiome by SARS-CoV-2 could contribute or exacerbate the gastrointestinal symptoms related to infection by other “classic” gastrointestinal pathogens.

In the last years, polymerase chain reaction-based assays have improved the ability to diagnose GIs due to their high sensitivity and specificity, and are hence routinely used in many clinical laboratories and hospitals [12].

Until now, no data on the potential correlation between diarrhoea-causing pathogens and SARS-CoV-2 infection are available. Therefore, this 2-years retrospective study aimed to evaluate the prevalence of “classic” gastrointestinal pathogens among SARS-CoV-2 infected and non-infected patients with diarrhoea referring to a University Hospital in Italy.

## 2 Material and methods

### 2.1 Sample collection

From February 1^st^^,^ 2022 to October 31^st^^,^ 2023, n. 833 patients admitted at Policlinico Ospedaliero-Universitario “Riuniti” (Foggia, Italy) for several admission diagnoses were enrolled in the study. The patients presented diarrhoea accordingly the definition as reported in ref. [13]. Out of 833 patients, n. 718 (86.2%) came from medical area, n. 68 (8.2%) from long-term care units, n. 18 (2.2%) from surgical area, n. 16 (1.9%) from traumatology area, n. 12 (1.4%) from emergency department and n. 1 (0.1%) from intensive care unit.

Each patient was subjected (either for screening or for suspected infection) to nasopharyngeal swab for Real Time reverse-transcription-polymerase chain reaction (RT-PCR) detection of SARS-CoV-2 and to stool swab for the research of the most common gastrointestinal pathogens by Real Time PCR test. Patients tested positive for SARS-CoV-2 were defined as “COVID-19 positive”.

The “Policlinico Riuniti” is a public, academic hospital that cares for a combined urban and suburban population of more than 594,000 citizens. Increasing immigrations rates, mainly from African countries and Eastern Europe, have been reported within this sanitary area in the last decade [14].

Demographic data (gender, geographical origin and age group) were obtained from the Hospital Information Management System. Clinical data (i.e., presence of gastrointestinal symptoms; eosinophilia; immunodeficiency and antibiotic treatments) were obtained from medical records.

Although data regarding duration of gastrointestinal symptoms in patients with COVID-19 are limited, a study found that COVID-19-positive patients with gastrointestinal symptoms alone had on average 16 days from initial symptom onset to hospital admission and 31 days from symptom onset to viral clearance [15]. Therefore, stool tests performed either 2 weeks before or 2 weeks after SARS-CoV-2 positivity were included in order to obtain an adequate time period during which patients may have had symptoms related to COVID-19.

### 2.2 SARS-CoV-2 PCR Panel Assay

All nasopharyngeal swab samples were subjected to RNA extraction by using MagCore® Nucleic Acid Extraction Kit (RBC Bioscience, Taiwan) and immediately subjected to real time RT-PCR screening by the Allplex™ SARS-CoV-2 Assay (Seegene Inc. Seoul, Korea) to determine SARS-CoV-2 infection**,** following the manufacturer's protocol.

### 2.3 Gastrointestinal pathogen PCR Panel Assay

All the stool swab samples were subjected to total nucleic acid (DNA/RNA) extraction by using MagCore® Nucleic Acid Extraction Kit (RBC Bioscience, Taiwan) and immediately subjected to multiplex real time PCR assay by the Allplex™ Platform (Seegene Inc. Seoul, Korea) in order to identify the causative pathogens of the gastrointestinal diseases. Allplex™ GI-Virus, Allplex™ GI-Bacteria and Allplex™ GI-Parasite (Seegene Inc. Seoul, Korea) assays were used to detect virus, bacteria and protozoan parasites, respectively (Table 1).

**Table 1:** Gastrointestinal pathogens (GPs) screened by Allplex™ Assays.

Allplex^TM^-GI-Virus	Allplex^TM^-GI-Bacteria	Allplex^TM^-GI-Parasites
*Astrovirus*	*Aeromonas* spp.	*Blastocystis* sp.
*Norovirus*	*Campylobacter* spp.	*Cryptosporidium* spp.
*Adenovirus*	*Clostridioidesdifficile* toxin B	*Cyclospora cayetanensis*
*Sapovirus*	*Salmonella* spp.	*Dientamoeba fragilis*
*Rotavirus*	*Shigella* spp.	*Giardia duodenalis*
	*Vibrio* spp.	*Entamoeba histolytica*
	*Yersinia enterocolitica*	
	*Escherichia coli* O157	
	Hypervirulent *Clostridioidesdifficile*	

The potential identified protozoan parasites were then genetically characterized as reported elsewhere for *Blastocystis* sp. [16], *Cryptosporidium* spp. and *G. duodenalis* [17], *C. cayetanesis* [18], *D. fragilis* [19] and *E. hystolytica* [20]. All the protozoan parasite isolates sequences were submitted to GenBank Database and the Accession Number reported in Table 5.

### 2.4 Statistical analysis

The statistical model was built to examine whether the gastrointestinal infections (overall and singular viral, bacteria and protozoan parasites infections) were associated with COVID-19 positivity. Gender (male vs female), origin (Western Europe, North Africa, Eastern Europe, East/Central Asia, South America) and age classes (0–18; 19–39; 40–59; 60–79; 80–99) were analysed as secondary variables. Statistical analysis was performed using the QuickCalcs GraphPad online tool (available at https://www.graphpad.com/quickcalcs/). The relationship between variables was examined by Chi-square test. A *p* value < 0.05 was considered statistically significant.

## 3 Results

### 3.1 Population description

Out of 833 enrolled patients, 395 (47.4%) were male and 438 (52.6%) were female. All patients were divided in five geographical origin groups: i) Western Europe (Italians, n = 796; 369 male and 427 female); ii) North Africa (n = 21; 17 male and 4 female); iii) Eastern Europe (Romanians; n = 14; 9 male and 5 female); iv) South America (n = 1; 1 female); and v) East/Central Asia (n = 1; 1 female).

Five age classes were defined as the following: i) 0–18 (n = 271, 32.5%), 19–39 (n = 64, 7.7%), 40–59 (n = 87, 10.4%), 60–79 (n = 186, 22.3%) and 80–99 (n=225, 27%). The mean age was of 47 ± 34.20 (age ranged from 0 to 99 years). All the associated demographics data are reported in Table 2.

**Table 2:** Demographics data (gender, origin and age classes) of the enrolled patients and of COVID-19 positive and negative patients.

Social demographic variables	Total cases (n = 833), (n, %)	COVID-19 positive (n = 81), (9.7%)	COVID-19 negative (n = 752), (90.3%)	*p* value
Age (mean ​+ ​−SD)	49 +-34.20	60 +-34.21	47 +-34.20	
Gender				
Male	395 (47.4%)	40 (49.4%)	355 (47.2%)	0.7095
Female	438 (52.6%)	41 (50.6%)	397 (52.8%)	
Origin				
Western Europe	796 (95.5%)	74 (91.3%)	722 (96%)	0.0535
North Africa	21 (2.5%)	5 (6.2%)	16 (2.1%)	0.0273^a^
Eastern Europe	14 (1.7%)	2 (2.5%)	12 (1.6%)	0.5612
South America	1 (0.1%)	0	1 (0.1%)	n.d.
East/Central Asia	1 (0.1%)	0	1 (0.1%)	n.d.
Age classes				
0–18	271 (32.5%)	14 (17.3%)	257 (34.2%)	0.0020^a^
19–39	64 (7.7%)	7 (8.6%)	57 (7.6%)	0.7331
40–59	87 (10.4%)	5 (6.2%)	82 (11%)	0.1859
60–79	186 (22.3%)	28 (24.7%)	158 (21%)	0.0054^a^
80–99	225 (27%)	27 (33.3%)	198 (26.3%)	0.1774

a *p* value: statistically significant.

### 3.2 Association between SARS-CoV-2 and gastrointestinal pathogens infection

Out of 833 patients, 81 (9.7%) were positive for SARS-CoV-2 PCR Panel Assay, while 752 (90.3%) were negative. The distribution of patients positive and negative to COVID-19 according the gender, origin and age classes is showed in Table 2. The positivity to COVID-19 were statistically associated with North Africa origin (*p* = 0.0273) and with age classes 60–79 (*p* = 0.0054), while no statistically significant correlation were observed with gender. By contrast COVID-19 negative patients were more frequently in the age classes 0–18 (*p* = 0.0020).

Amid the COVID-19-positive patients, thirty (n = 30/81; 37%) were found positive for one or more gastrointestinal pathogens, with a higher prevalence of protozoan parasites (18.5%) followed by bacterial (7.4%) and viral (4.9%) pathogens (Table 3). Regarding COVID-19-negative patients, 191 patients (n = 191/752; 25.4%) were found positive to one or more gastrointestinal pathogens, with a higher prevalence of viral pathogens (14.8%) followed by bacterial (6.9%) and protozoan parasites (2.6%) (Table 3). Simultaneous presence of multiple pathogens (i.e., virus/virus, virus/bacteria, virus/protozoa, bacteria/bacteria, bacteria/protozoa and protozoa/protozoa) was reported in 6.2% and 1.1% of patients positive and negative to COVID-19, respectively (Table 3).

**Table 3:** Viruses, bacteria and protozoan parasites (singular and mixed infection) detected among COVID-19 positive and negative patients.

Gastrointestinal pathogens group	COVID 19 positive patients (n = 81)	COVID 19 negative patients (n = 752)	*p* value
VIRAL PATHOGENS			
*Adenovirus*	3 (3.7%)	46 (6.1%)	
*Norovirus*	1 (1.2%)	36 (4.8%)	
*Sapovirus*	0	12 (1.6%)	
*Astrovirus*	0	11 (1.5%)	
*Rotavirus*	0	6 (0.8%)	
**TOT.**	**4 (4.9%)**	**111 (14.8%)**	**0.0149**^a^
**BACTERIAL PATHOGENS**			
*Campylobacter* spp.	4 (4.9%)	34 (4.5%)	
*Salmonella* spp.	2 (2.5%)	11 (1.5%)	
*Aeromonas* spp.	0	6 (0.8%)	
*Yersinia enterolitica*	0	1 (0.1%)	
**TOT.**	**6 (7.4%)**	**52 (6.9%)**	**0.8686**
**PROTOZOAN PARASITES**			
*Blastocystis* sp.	13 (16%)	8 (1.1%)	
*Dientamoeba fragilis*	2 (2.5%)	4 (0.5%)	
*Giardia duodenalis*	0	4 (0.5%)	
*Cryptosporidium* spp.	0	4 (0.5%)	
**TOT**	**15 (18.5%)**	**20 (2.6%)**	**0.0001^b^**
**MIXED PATHOGENS**			
*Adenovirus/Astrovirus*	0	1 (1.2%)	
*Adenovirus/Campylobacter*	0	1 (1.2%)	
*Norovirus/Aeromonas*	0	1 (1.2%)	
*Sapovirus/Salmonella*	0	1 (1.2%)	
*Sapovirus/Dientamoeba*	0	1 (1.2%)	
*Norovirus/Giardia*	0	1 (1.2%)	
*Campylobacter/Blastocystis*	1 (1.2%)	0	
*Clostridium/Blastocystis*	2 (2.5%)	0	
*Blastocystis/Dientamoeba*	1 (1.2%)	1 (1.2%)	
*Blastocystis/Dientamoeba/Giardia*	0	1 (1.2%)	
*Dientamoeba/Blastocystis/Adenovirus*	1 (1.2%)	0	
**TOT**	**5 (6.2%)**	**8 (1.1%)**	**0.0004^b^**
**Overall**	**30 (37%)**	**191 (25.4%)**	**0.0242**^a^

a *p* value: statistically significant; ^*b*^*p* value: highly statistically significant.

Considering the single group of pathogens, a highly statistically significant correlation was observed between COVID-19 positivity and presence of protozoan parasites (*p* = 0.0001) and multiple pathogens (*p* = 0.0004) (Table 3). Conversely, a statistically significant correlation was observed between COVID-19 negativity and viral pathogens (*p* = 0.0149). The association between COVID-19 and presence of bacterial pathogens was not significant (Table 3).

Overall, the association between COVID-19 positivity and gastrointestinal pathogens (all types) was statistically significant (*p* = 0.0242) (Table 4).

**Table 4:** Number of patients (and relative percentage) positive and negative to one or more gastrointestinal pathogens (GPs) and with/without SARS-CoV-2 infection (COVID-19 positive/negative).

Total population (n = 833)	GPs positive (n. %)	GPs negative (n.%)	*p value*

COVID-19 positive (n. 81, 9.7%)	30 (37%)	51 (63%)	0.0242^a^
COVID-19 negative (n.752, 90.3%)	191 (25.4%)	561 (74.6%)	

### 3.3 Genetic characterization of protozoan parasites

Following the molecular characterization of isolated protozoan parasites, four subtypes (ST1-ST4) were identified for *Blastocystis* sp., Assemblages A and A1 for *G. duodenalis*, genotype IIa for *C. parvum* and genotype 1 for *D. fragilis* (Table 5).

**Table 5:** Number of subtypes/assemblages/genotypes and sequence Accession Number of protozoan parasites isolated in COVID-19-positive and COVID-19 negative patients.

Protozoan parasites	COVID-19-positive patients	COVID-19-negative patients	Accession Numbers
*Blastocystis* sp.			
*Blastocystis* ST1	4	2	OR936065, OR936069, OR936071, OR936072, OR936082, OR936083
*Blastocystis* ST2	2	1	OR936080, OR936088, OR936090
*Blastocystis* ST3	6	3	OR936066, OR936076, OR936077, OR936081, OR936084, OR936085. OR936086, OR936089, OR936091
*Blastocystis* ST4	1	2	OR936069, OR936075, OR936092
***Dientamoeba fragilis***			
*Dientamoeba fragilis* genotype I	2	4	OR921928, OR921929, OR921930, OR921931, OR921932, OR921933
***Giardia duodenalis***			
Assemblage A	0	1	OR941425
Assemblage A1	0	3	OR941427, OR941428, OR941429
***Cryptosporidium* spp**			
*Cryptosporidium parvum* IIa	0	4	OR941421, OR941422, OR941423, OR941424
***Mixed infections***			
*Sapovirus/D. fragilis* genotype I	0	1	OR921934
*Norovirus/G. duodenalis* Assemblage A	0	1	OR941426
*Campylobacter/Blastocystis* ST2	1	0	OR936078
*Clostridium/Blastocystis* ST3	2	0	OR936067, OR936074
*Blastocystis* ST2*/D. fragilis* genotype I	1	0	OR921935, OR936070
*Blastocystis* ST1*/D. fragilis* genotype I	0	1	OR921936, OR936087
*Blastocystis* ST2*/D. fragilis* genotype I*/G. duodenalis* Assemblage A1	0	1	OR941430, OR921937, OR936079
*D. fragilis genotype I/Blastocystis* ST3*/Adenovirus*	1	0	OR921938, OR936073

## 4 Discussions

Nowadays gastrointestinal disease still remains a significant global health concern. Indeed, according to the World Health Organization, there are 1.7 billion total cases each year, causing approximately 750,000 deaths mainly among children younger than 5 years old [3]. Gastrointestinal infections caused by bacteria (i.e., *Clostridioides difficile*, *Escherichia coli*, *Shigella*), viruses (i.e., norovirus, rotavirus), or protozoan parasites (i.e., *Cryptosporidium*, *Giardia*) are major causes of diarrheal illness worldwide, often resulting from contaminated food/water and poor sanitation [21]. Disruption of normal mucosal defences by one enteric infection is recognized as a risk factor for other infections, and prior studies have found that up to 26% of patients undergoing stool pathogen testing carry multiple pathogens [5].

It is known that among patients positive to COVID-19, the most common manifestations include fever, dry cough, dyspnoea, weakness, breathing difficulty, fatigue, and myalgia [22]. However, some patients also present gastrointestinal symptoms including nausea, vomiting, diarrhoea, and abdominal pain. Within patients suspected for COVID-19 infection, gastrointestinal symptoms could be the initial presenting symptoms [23]. Some patients may present only gastrointestinal symptoms during the disease, which can delay the diagnosis leading hence to potential complications for themselves and infection transmission to others [23]. Previous studies have indicated that SARS-CoV-2 enters cells through the ACE2 receptors expressed within the human respiratory and gastrointestinal (oesophagus, ileum, and colon) tracts. The ACE2 receptors in the gastrointestinal tract maintain a regulatory role in amino acid homeostasis, gut microbiome, and innate immunity [24]. Consequently, the binding of SARS-CoV-2 to ACE2 receptors in the gastrointestinal tract may result in a dysbiosis with gastrointestinal symptoms such as abdominal pain and diarrhoea [23]. In addition, it is possible that this intestinal dysbiosis and inflammation due to SARS-CoV-2 infection could predispose individuals to co-infection with other gastrointestinal pathogens that can be assumed by oral-faecal route.

In this retrospective study, a potential interaction between COVID-19 positivity and the presence of “classic” gastrointestinal pathogens in patients with diarrheal disease has been investigated.

Out of 833 diarrheic patients and subjected to SARS-CoV-2 test, 9.7% (81/833) resulted positive and 90.3% (752/833) resulted negative to COVID-19. Although the SARS-CoV-2 infection was more frequent in male patients, any statistically significant correlation has been found between COVID-19 positivity and gender. By contrast, a statistically significant correlation has been found between COVID-19 positivity and patients come from North Africa (unfortunately, we are not able to know if the infection was acquired in Italy or in their country of origin) and in elderly people (age group 60–79 years), as expected.

In our work, an association statistically significant (*p* = 0.0242) has been found between COVID-19 positivity and gastrointestinal pathogens (all types); in particular protozoan parasites were the most detected gastrointestinal pathogen group (18.5%) following by bacterial pathogens (7.4%) and mixed infections (6.2%). Viral pathogens (14.8%) were more frequent among COVID-19 negative patients.

Amongst protozoan parasites, a higher prevalence of *Blastocystis* sp. (n = 13, 16%) was reported within COVID-19-positive subjects when compared to the COVID-19-negative patient prevalence (n = 8, 1.1%). This result (although with a higher prevalence) is in accordance with a recent study conducted in an Hospital in Teheran, Iran, that focused on studying the frequency of intestinal parasitic infections within a cohort of COVID-19 positive patients and reporting hence a *Blastocystis* sp. prevalence of 6% [25]. In another study conducted in Arabia Saudia, a SARS-CoV-2 and *Blastocystis* sp. mixed infection was reported in a woman with a congenital chloride losing diarrhoea (CCLD) [26] leading the authors to hypothesize that the two microorganisms could have exacerbated the osmotic diarrhoea typical of CCLD [27]. Although the pathogenic role of *Blastocystis* sp. remains still controversial since this protozoan parasite has been found in healthy and non –healthy patients [28,29], the last literature data showed the potential ability of *Blastocystis* sp. to alter the gut microbiota ecosystem, which may lead to beneficial or harmful functions in the digestive system [29]. This gut microbiome alteration could predispose individuals to co-infection with other gastrointestinal pathogens and favour or exacerbate also the inflammation due to SARS-CoV-2 infection. Based on the results here obtained a potential interaction between SARS-CoV-2 and *Blastocystis* sp. could be then hypothesized.

By genotyping our *Blastocystis* sp. isolates, subtypes from ST1 to ST4 were identified, with ST3 the most prevalent subtype, as expected [16]. Actually, among thirteen *Blastocystis*-COVID-19 positive patients, subtype ST3 was detected in six patients, following by ST1 in four patients, ST2 in two patients and ST1 in one patient. The higher number of ST3 subtypes were also reported in a similar study carried out in a hospital in Teheran, in which a high prevalence of *Blastocystis* sp. has been found in COVID-19-positive patients with ST3 being the most common detected subtype [30,31]. Nevertheless, these results prone the basis for future investigations focusing on the association between *Blastocystis* and COVID-19-positive samples in order to establish potential risk factors for gastrointestinal symptoms.

In our work, *D. fragilis* was found in 2.5% of patients with COVID-19 when compared with those COVID-19 negative (0.5%). Similar to *Blastocystis* sp., the pathogenic role of *D. fragilis* is still controversial but several studies reported a cooperation between the two protozoan parasites [32]. Indeed, here, a mixed infection *Blastocystis* sp./*D. fragilis* were reported in two patients COVID-19 positive patients. Interestingly, all our *D. fragilis* isolates have been genotyping as Genotype 1 variant that being the most reported and pathogenic genotype [33,34].

Quite unexpected was the detection of *C. parvum* genotype IIa (n. 4, all Italian origin) and *G. duodenalis* Assemblages A and A1 (n. 4, all Italian origin) (the most frequently protozoan parasites causing diarrhoea and with a high zoonotic impact) only within our COVID19-negative patients whereas the presence of *C. parvum* IIa and *G. duodenalis* was observed in COVID-19 positive patients from a study carried out in Teheran, although with a very low prevalence [25]. In these patients group any correlation with origin, gender or age were found and mostly probably the diarrhoea was related just to parasite infection.

Among bacterial pathogens, *Campylobacter* sp. and *Salmonella* sp. were the only bacteria species found in COVID-19-positive patients with prevalence of 4.9% and 2.5%, respectively although without any statistically significant correlation. Interestingly, mixed infections *Campylobacter*/*Blastocystis* and *Clostridioides/Blastocystis* were reported in one and two COVID-19 positive patients, respectively, with a statistically significant association. In a recent work analysing the occurrence of *Blastocystis* sp. in patients with *C.* *difficile* in a cohort study from Colombia, the authors demonstrated a significant association between the presence of *Blastocystis* and *C.* *difficile* infection (CDI), with 61 cases of co-infection among CDI patients. This co-infection could support hypotheses that *Blastocystis* may adapt to dysbiosis and oxidative stress by *C.* *difficile* and together exacerbated the gastrointestinal symptoms [35].

Concerning viral pathogens, although Adenovirus and Norovirus were reported in the 3.7% and 1.2% of COVID-19-positive patients, we found a statistically significant association between COVID-19 negative patients and presence of viral pathogens (*p* = 0.0139). This result was quite unexpected since previous reports stating that the circulation and morbidity burden of other respiratory viruses (including adenoviruses) are also highly impacted by the COVID-19 pandemic, with wide temporal and geographic fluctuations [36]. Conversely, a mixed infection *Adenovirus/Blastocystis/Dientamoeba* was registered in one COVID-19 positive patient.

In this study we found that patients with COVID-19 were more likely to test positive for co-infection with classic gastrointestinal pathogens compared to those without COVID-19, although any correlation with gender, origin or age group has been found. This difference was mainly driven by protozoan parasites (highly statistically significant) followed by mixed infection (statistically significant). Although the majority of gastrointestinal symptoms in COVID-19 patients may be attributable to viruses, our results show that the most commonly identified enteric infections were related to protozoan parasites species, which was unexpected given these are relatively neglected pathogens. Based on the literature reports, intestinal protozoa can potentially polarize the helper T cells towards type 1 (Th1) [25]. Additionally, co-infections with intestinal protozoa and some intracellular pathogens, such as *Mycobacterium tuberculosis* and human immunodeficiency virus (HIV), may substantially cause imbalances in the host and lead hence to further pathological consequences [37]. Since the emergence of the SARS-CoV-2, there have been some hypotheses on the likely interaction between the intestinal parasites and COVID-19 [38]. Nevertheless in COVID-19 positive patients investigated in the present study, diarrhoea cannot be reliably attributed solely to the *Blastocystis* sp., because other infectious (bacterial and viral agents) and/or non-infectious diseases may have had a role in the initiation and progression of diarrhoea. For this reason, in order to establish the existence of a probable correlation between the *Blastocystis* sp. and SARS-CoV-2 infection, more deeply studies are needed. In particular, as our next goal, a prospective study on a large scale, regarding the analysis of the composition of gut microbiota and its correlations with different risks factors for each patient with gastrointestinal diseases will be designed. This will allow us to better understand the role of protozoan parasites and in particular for *Blastocystis* sp. in a scenario of intestinal dysbiosis and try to elucidate the modulating role of this eukaryote on the members of the bacterial microbiome, particularly in those cases of co-infection with SARS-CoV-2.

## 5 Study limitations

To the best of our knowledge, this is the first study, conducted over a period time of almost two years, reporting the prevalence of co-infection with gastrointestinal pathogens using PCR-based assays in patients under evaluation for COVID-19 infection. However, the present study has some limitations, mainly due to its retrospective design. In particular, we have had not the change to distinguish the severity of diarrhoea and therefore the differences between groups. In addition, we were not able to accurately retrieve the medical history for each patient (i.e. travel to other countries, the time between the arrival in Italy and clinical manifestation) and evaluate clinical features such as patients’ immunological conditions and haematological parameters.

## Ethics approval and consent to participate

The present study was performed following the guidelines of the Declaration of Helsinki in 1975, revised in 2013 and all the procedures performed in this study meet the national and international guidelines. Ethical Committee approval was obtained with number 10/CE/2024 of January 31, 2024. Included samples were obtained according to standard diagnostic and therapeutic protocols for the management of gastrointestinal infections. All the authors ensure that this study is HIPAA (Health Insurance Portability and Accountability Act, 1996) compliant. The researchers followed every mandatory (health and safety) procedure.

## Consent for publication

Not applicable.

## Availability of data and materials

Not applicable.

## Funding

The authors declare that no specific funding was used for this research.

## CRediT authorship contribution statement

**Marianna Marangi:** Conceptualization, Data curation, Formal analysis, Investigation, Methodology, Supervision, Validation, Visualization, Writing – original draft, Writing – review & editing. **Sonia Boughattas:** Methodology, Writing – review & editing. **Felice Valzano:** Writing – review & editing. **Gianfranco La Bella:** Writing – review & editing. **Rosella De Nittis:** Visualization. **Maurizio Margaglione:** Visualization. **Fabio Arena:** Writing – review & editing.

## Declaration of competing interest

The authors declare that they have no conflict of interest and that they have no actual or potential competing financial interests.
